# Barn-Raising on the Digital Frontier: The L.A.U.N.C.H. Collaborative

**DOI:** 10.13023/jah.0201.02

**Published:** 2020-01-26

**Authors:** Bradford W. Hesse, David Ahern, Michele Ellison, Eliah Aronoff-Spencer, Robin C. Vanderpool, Karen Onyeije, Michael C. Gibbons, Timothy W. Mullett, Ming-Yuan Chih, Victoria Attencio, Grant Patterson, Jessica Boten, Christopher Hartshorn, Ben Bartolome, Katie Gorscak, Melanie McComsey, Alexandra Hubenko, Bin Huang, Corey Baker, Don Norman

**Affiliations:** National Cancer Institute, hesseb@mail.nih.gov; Federal Communication Commission, david.ahern@fcc.gov; Federal Communications Commission, Michele.Ellison@fcc.gov; University of California San Diego, earonoffspencer@ucsd.edu; NIH, vanderpoolrc@mail.nih.gov

**Keywords:** Appalachia, connected health, implementation science, broadband connectivity, rural health, cancer, communication

## Abstract

A meta-analysis of oncology papers from around the world revealed that cancer patients who lived more than 50 miles away from hospital centers routinely presented with more advanced stages of disease at diagnosis, exhibited lower adherence to prescribed treatments, presented with poorer diagnoses, and reported a lower quality of life than patients who lived nearer to care facilities. Connected health approaches—or the use of broadband and telecommunications technologies to evaluate, diagnose, and monitor patients beyond the clinic—are becoming an indispensable tool in medicine to overcome the obstacle of distance.

## SOLVING THE LAST-MILE PROBLEM IN APPALACHIA

A recent meta-analysis of oncology papers from around the world revealed that cancer patients who lived more than 50 miles away from hospital centers routinely presented with more advanced stages of disease at diagnosis, exhibited lower adherence to prescribed treatments, presented with poorer diagnoses, and reported a lower quality of life than patients who lived nearer to care facilities.^1^ These findings come as no surprise to researchers dedicated to improving the health of Appalachian communities; these research teams have documented similar findings for patients living in rural, underserved communities in this unique geopolitical region.^2^–^4^ In fact, epidemiologists have noted that while national age-adjusted mortality rates for cancer have been falling steadily since 1993, progress has stalled in nonmetropolitan communities where access to preemptive cancer screening services and close vigilance during treatment is a challenge.^5^–^7^

Connected health approaches—or the use of broadband and telecommunications technologies to evaluate, diagnose, and monitor patients beyond the clinic—are becoming an indispensable tool in medicine to overcome the obstacle of distance.^8^ However, access to, and adoption of, the necessary broadband and telecommunications infrastructure needed to support connected health approaches remains a problem for widespread implementation. Simply stated, broadband is not equally available across the United States. Within the telecommunications industry, completing this last leg of connectivity is commonly referred to as *solving the last mile problem*, or as leaders at the Federal Communications Commission (FCC) suggest *bridging the digital divide*.^9^,^10^ Healthcare has an equivalent last mile problem, we would argue, which complicates health system planning as administrators seek to ensure that the benefits of hard-won medical knowledge are equitably distributed across all populations, regardless of ZIP code.^11^ As medicine goes digital, solving the last mile problem for the nation’s telecommunications infrastructure will become a necessary precondition for solving the last mile problem in healthcare. The stakes are high for solving these problems concurrently. In the case of cancer, analysts from the American Cancer Society have suggested that up to 22% of the more than half-million lives we expect to lose from cancer annually could be saved by providing equitable access to evidence-based knowledge and its subsequent practice across all populations.^12^

## CONNECTING HEALTH

In 2016, the President’s Cancer Panel—a legislatively mandated advisory committee delivering periodic assessments on the state of the National Cancer Program to the President of the United States—released a report titled “Improving Cancer Outcomes through Connected Health.”^13^ The report enumerated the ways in which the strategic application of digital health technologies were beginning to address several of the patient or provider access issues that had been hampering patient outcomes in contemporary oncology. For example, the report presented the example of how a large health maintenance organization in Southern California had been able to use its Electronic Health Record (EHR) system to keep tabs on the progress of its individual members in meeting the screening recommendations suggested by the U.S. Preventive Services Task Force. Based on that information, digital prompts were triggered sending outreach messages to patients and office staff alike. Electronic dashboards kept track of every patient in the system, prompting follow-up when needed to encourage compliance proactively per newly re-scripted protocols. When reviewed through external evaluations, the approach yielded substantive improvements in screening rates equitably across all populations with no sign of disparity.^14^,^15^ In other examples, remote monitoring systems designed to integrate patient-generated data either through personal reports or digital sensing devices (e.g., on patient or ambient) were shown to be effective in averting adverse reactions to treatment, in preventing unnecessary hospitalizations, in improving outcomes (including survival rates), and in elevating patients’ quality of life.^16^,^17^

The report came with a caveat, however. According to data collected by the FCC and analyzed by the Connect2Health^FCC^ Task Force^a^, access to broadband connectivity for health appeared to be unevenly distributed across the country. Some counties, especially counties in rural areas, suffered from significant broadband access, as well as adoption gaps. Paradoxically, these were the very counties that would benefit the most from the broadband-enabled health solutions that were being used to foster continuous care approaches and elevate health outcomes in other parts of the country.^18^ Indeed, the data suggested that the picture of health was vastly different in “connected” communities and “digitally-isolated” communities—a finding that held true across the access to care, quality of care and health outcome metrics that were studied.^19^ The Connect2Health^FCC^ Task Force reasoned that this lack of access to high-speed Internet connectivity was likely contributing to a lack of progress in preemptively averting the consequences of diseases such as diabetes, cardiovascular disease, addiction, and cancer. In public health terms, a lack of access to broadband, and the health information infrastructure it enables, was rapidly becoming a social determinant of health—if not a “super” determinant of health—for individuals living within these counties.^20^,^21^ Specifically, and recognizing the public policy implications of these findings, the Connect2Health^FCC^ Task Force examined the relationship between the level of connectivity in a community and that community’s health, and whether increasing broadband connectivity correlates to improved health outcomes at the community and populations levels. This data analysis found a persistent relationship at the population level (albeit not causal) between lower diabetes prevalence (a health outcome) and higher levels of broadband connectivity; and this was the case even after controlling for potentially confounding factors like income, age, or rurality. The President’s Cancer Panel report recommended that representatives from the National Cancer Institute (NCI) work directly with initiatives such as the Connect2Health^FCC^ Task Force to facilitate health information access and sharing by ensuring adequate Internet access.

## THE L.A.U.N.C.H. COLLABORATIVE

As this dialogue began, the FCC and NCI soon realized that independently each agency was tackling the problem of improving rural health care from different but complementary ends of the spectrum. The FCC had been exploring a multi-stakeholder initiative in Appalachia that would leverage broadband to create an “ecosystem of care” around rural patients not just around the clinical encounter (i.e., connecting a patient with cancer to a specialist), but also leveraging cutting-edge technologies like sensors (in the home and on the patient) along with artificial intelligence (AI) and mHealth technologies to bridge gaps created by time and distance while promoting actionable intelligence for clinical care teams, integration with the EMR or other data repositories, and increased patient engagement, all at lower cost. NCI was similarly exploring innovative ways to improve access to digital tools and capacity for cancer care, as contemplated by the President’s Cancer Panel Report.

In December of 2017, the FCC and the NCI leadership signed a Memorandum of Understanding (MOU) formally declaring their intention to work collaboratively in identifying the ways in which rural communities could benefit from improved access to broadband health.^9^ In taking this visionary step, NCI Director Ned Sharpless tweeted that the “L.A.U.N.C.H. Collaborative [will] increase broadband access and can help make a difference to cancer patients in rural Appalachia.”^22^ Similarly, FCC Chairman Ajit Pai reiterated the FCC’s commitment to help “address the broadband health gap in Appalachia.”^23^ More recently, Chairman Pai emphasized that “connecting communities and health systems through deployment of high-speed broadband is essential to improving our nation’s health,” adding that “cancer represents a particularly compelling use case for the power of connectivity to reduce the burden of disease in our rural communities.”^24^

Indeed, data from the national cancer registries suggested that one of the geographic areas in greatest need of improvement was in the Appalachian region where deaths from lung and many other cancers top the nation in number and severity.^3^,^5^ This is also an area of the country that data from the FCC showed to be underserved by broadband, leading to a comparatively high frequency of “double-burden” counties^18^—that is, counties that were manifesting both lower rates of broadband access and Internet adoption (including for digital health technologies) and high burdens of disease. With these trends as background, the NCI and the FCC partnered with the NCI-designated University of Kentucky (UK) Markey Cancer Center (MCC) to serve as a vanguard for demonstration efforts intended to improve cancer-related outcomes and enhance care for patients living in underserved areas, such as Appalachia, through broadband-enabled, connected health solutions.

In addition to its recommendation for improving population access to digital capacity, the President’s Cancer Panel encouraged activity that would (a) improve the interoperability of data flows between connected health devices, EHRs, and healthcare systems; (b) address usability issues to reduce burden and improve uptake; and (c) engineer solutions in support of an oncology workforce struggling to stay abreast of the increasing demand from an aging population.^13^ In response to these recommendations, NCI also partnered with The Design Lab at the University of California San Diego (UCSD), a national leader in human-centered design and creator of sustainable, usable systems in healthcare. Finally, as a result of its history of collaboration with the UCSD Design Lab, the public–private partnership arm of Amgen also joined the larger Collaborative, bringing to the table expertise in formative evaluation and customer engagement. A key goal of this founding Collaborative was to demonstrate the value of broadband-enabled health approaches in hard-to-reach geographies and to set the stage for future scalability and new partner involvement.

Working together, the newly formed Collaborative developed a proposal for a 3-year demonstration project designed to foster improvement in cancer outcomes through a Collaborative termed “Linking and Amplifying User-Centered Networks through Connected Health,” or L.A.U.N.C.H. for short. With the transformative power of broadband in our collective sights, our vision was to catalyze a new era in connected cancer care for Appalachia.^25^ In this regard, the Collaborative sought not just to connect rural and remote cancer patients to state of the art clinical care available in more urban areas, but also to identify novel and effective ways of monitoring distress and meeting the needs of rural cancer patients; connectivity presented a unique opportunity to develop a model that was real-time, adaptive, and designed to promote several of the Cancer Panel’s goals around interoperability, usability, and workforce demands, potentially alongside social determinants of health.^b^

At the core of the project was an innovative methodology proposed by the UCSD Design Lab’s Director Don Norman, a leader in the area of user-centered design for more than three decades. In a précis titled “Community-Based, Human-Centered Design,” he offered a proposal to the L.A.U.N.C.H. Collaborative on how to promote successful implementation of human-centered design principles, community-by-community, at scale.^26^ The key concept that the L.A.U.N.C.H. Collaborative ultimately adopted was to move away from a top-down model historically popular in medicine where experts laboriously consume time and resources translating knowledge into practice for local benefit, but rather to provide the resources to communities so that they can work with resident experts in co-designing superior solutions for themselves.

This collaborative consumer-expert concept, for good reason, reflected emerging principles from the “platform revolution”^14^ that are disrupting businesses in almost every other sector of the economy. Principles from a platformed business approach call for a flip in traditional, industrial-age business models. Rather than provide value by way of top-down, expert support for local customers—a practice that requires maintenance of expensive pipeline for service distribution—many information-age businesses are providing value by creating the platform, and curating the relationships, needed for consumers to work together in creating their own solutions locally. This is the reason why companies such as Google, Amazon, Airbnb, Uber, and others have all risen precipitously in market valuation; the model scales more quickly than top-down, pipeline approaches. By providing a platform for agile development (referred to as our L.A.U.N.C.H.-PAD), rather than incurring the costs and time needed to create a centralized supply chain, the L.A.U.N.C.H. Collaborative will accelerate implementation by giving communities the tools to solve last mile problems in parallel. The approach took its cues from a National Cancer Advisory Board (NCAB) Blue Ribbon Panel Report on the “Cancer Moonshot^SM^” initiative.^27^ The spirit of the Cancer Moonshot^SM^ initiative, later funded through passage of the 21st Century Cures Act,^28^ was to accomplish in five years what would otherwise take ten. To do this, the NCAB committee encouraged the creation of patient-engagement networks and shared data flows to support advancement in oncologic medicine. It also emphasized the importance of accelerating the pace of implementation science so that more people would benefit from the knowledge already gained in the fight against cancer.^c^

The co-design, community-focused concept of L.A.U.N.C.H. seemed especially well-suited for a pilot study in rural Appalachia, where local independence and community partnerships are strong and impactful. As the operational director of the project Eliah Aronoff-Spencer observed: “this is a community that has been exemplary in its sense of self-reliance and creative problem-finding; just think about ‘barn-raising’ where community members would gather together to help an individual member of their community prepare for the harvest.” A blog posted on the NCI’s Implementation Science website described the approach as a marriage between design science, popular in engineering and industry, and community-based participatory research, popular in public health.^29^

## THE KENTUCKY PROJECT

As a trial balloon for this new approach to building capacity and resilience in our rural communities and to creating a new service model leveraging connectivity, the L.A.U.N.C.H. Steering Committee selected an implementation objective in Kentucky that had a prior evidentiary base for effectiveness, but that had been slow in achieving clinical uptake. The Steering Committee selected findings cited by the American College of Surgeons’ Commission on Cancer suggesting that distress screening during treatment and postoperative recovery can, and should, lead to improved patient outcomes.^30^,^31^ Such programs have been difficult to scale nationally, though, and could therefore benefit from an accelerated implementation science approach.^32^ At the same time, these same approaches are also especially well-suited for addressing the communication and coordination problems that are endemic to rural communities.

Once the geographic area for the pilot project had been determined, the L.A.U.N.C.H. Collaborative set out to design a staged protocol for achieving the objectives of a community-driven design effort. The Collaborative proposed a project plan broken into six phases based on best practice in design science.

1. **Phase 1: Learn**. During the learning phase, the L.A.U.N.C.H. team commissioned a qualitative ethnography team and a quantitative data analytic team to begin conducting formative research exploring topics such as: rural health and process disparities, broadband and information technology gaps, barriers and innovations in cancer symptom management, and identifying community assets for bolstering connected health programs.
2. **Phase 2: Listen**. While the learning phase allowed for passive observation, the listening stage was explicitly designed to enable members of the research team to embed themselves within the community to learn firsthand about patients’ deeply held values; to understand communities’ ongoing efforts to support better communication and better health (which included meetings with broadband and telecommunications providers as well as healthcare providers and public health practitioners); and to understand families’ support networks, needs, and attitudes toward ameliorating distress during serious illness. In addition to listening at the local level, the Collaborative sought to understand barriers and facilitators across multiple sectors from a national perspective.^24^ Borrowing from the scholarship on sustainable development and building cross-sectoral partnerships^33^, in May of 2019, the FCC and the NCI convened a Think Tank-styled meeting at FCC headquarters in Washington DC, with senior thought leaders from both the public and private sectors and across the country. This vehicle was carefully chosen to ensure that cross-fertilization and cross-pollination could occur across the sectors and in real-time, and to ensure a full understanding of the dynamics of the various business cases at play. The broad expertise included representatives from government, academia, industry, healthcare systems, public health, biotechnology, design and innovation, and telecommunications.^24^
3. **Phase 3: Co-Create**. A fundamental hypothesis of the L.A.U.N.C.H. project is that local implementation will work best if it is designed and guided by local stakeholders and beneficiaries of the system. During the co-creation phase, design experts worked directly with patients and caregivers through locally convened “Innovation Studios.” Within the studios, community teams worked to identify the core functionalities of a redesigned symptom management healthcare service, identified the workflows needed to tie assessments with action, identified ways in which broadband service could be extended to underserved areas in support of distress monitoring, and began assembling low-fidelity prototypes of proposed functional systems.
4. **Phase 4: Pilot**. Following the principles of agile design and transparent development as highlighted through the 21st Century Cures Act,^20^ the principal investigators began working with the MCC team to “launch” a series of self-improving, iterative pilot studies built on the specifications identified in Phase 3. An agreed-upon set of outcome measures was identified as the metric by which the pilots would be judged along with a protocol to ensure that all improvements to the care process would serve to improve (and not weaken) patient safety compared to standard of care. The protocols were approved through the relevant Institutional Review Boards to ensure adherence to Good Clinical Practice for a human factors-oriented, quality improvement study.
5. **Phase 5: Improvement**. Unlike traditional clinical trials to measure efficacy, where empirical control and nonvariation is the order of the day, design trials are derived from a human factors tradition and are designed to reduce the variance between the technological and human control systems over successive iterations.^21^ In the improvement phase, the community-led design teams were expected to evolve the usefulness of their systems through feedback from the pilots, continued field research, and co-creation sessions. Additionally, collaboration with national and local broadband providers would allow patients who do not have broadband access to obtain the service, thereby enabling a feedback loop with MCC as well as curated connections to appropriate community supports and services. As in other types of healthcare system design efforts, the goal is to iterate toward the quadruple aim of healthcare redesign: i.e., (a) improving the health of populations, (b) enhancing the experience of patients, (c) reducing per capita costs, and (d) reducing burden and enhancing joy for professional staff.^34^,^35^
6. **Phase 6: Scale**. If the demonstration project is successful, we would expect to fulfil at least three important outcomes. First, we would begin to see a paradigm of “community-based, human-centered design (2.0)” take hold in rural Appalachia. Within such a paradigm, we would expect to see local clinical and public health programs continue to work jointly with community planners and communications providers to close the digital divide in rural areas and to continue their progress in solving the last mile problem in oncology care. Second, we would hope to deliver a scalable platform, enabled by telecommunication and technology industry partners, to support community-led development of health interventions nationally. And third, this public-private project would help to further demonstrate the critical importance of increasing broadband access and adoption in the provision of health care—part of the FCC’s current policy priority of bridging the digital divide throughout the country, and especially in rural and underserved areas—and in improving cancer outcomes. Such a platform would not represent a top-down approach from federal agencies, but would help identify the toolkits, shared resources, and curated partnerships necessary for local innovations “launched” in parallel across the country.

## PARTNERING WITH THE *JOURNAL OF APPALACHIAN HEALTH*

Members of the L.A.U.N.C.H. Steering Committee recognized the unique opportunity to promote the work locally after editors of the newly commissioned *Journal of Appalachian Health (JAH)* reached out to gauge interest in publishing results from the project as the data become available. Indeed, the purpose of the demonstration project was to engage directly with community members in a spirit of collaboration and in service to local goals. Partnering with the *JAH* seemed to be a perfect opportunity to build on those ideals. It embodies our view of open, collaborative science; and it helps elevate the conversation in such a way that others can contribute knowledge and resources as we work together to solve the problem of limited access to proactive cancer care in rural Appalachia.

As we write this editorial, L.A.U.N.C.H. is entering its third year of the demonstration project. Much of the preliminary work we conducted during the “learning” and “listening” phases of the project has come to completion and will be presented in the first issue of *JAH*–L.A.U.N.C.H. series. This includes an extensive ethnographic study prepared as a technical report in background to the pilot study. Given that the journal is an open-access, online-only publication, the publishers have graciously offered to provide electronic copies of this report freely to their readers. A streamlined, peer-reviewed synopsis of this report will also be included along with a relevant blueprint for how lessons learned could influence ongoing implementation efforts in the digital health space. A preparatory literature review, summarizing the implications of the symptom management literature for researchers in rural Appalachia, will also be included, as will a quantitative analysis of double-burden counties falling out of the reach of medical care and the extensible capacities of broadband coverage.

Once the pilots are underway, data will become available to document the successes and/or challenges of deploying digital solutions for symptom management in Appalachian Kentucky. Our intention will be to provide ongoing analyses of these data to the *JAH* readership as the various phases of the demonstration project reach their conclusion. Where possible, we will strive to enrich those analyses with multimedia content to document the processes and results that would serve best to foster replication and improvement. As the year concludes, the Collaborative will endeavor to provide summary analyses, along with links to the finalized blueprints and toolkits, as a contributing resource to the *JAH*.

## CONCLUSION

In 2012, the National Academy of Medicine reported on a workshop in which one of the prevailing themes was that despite 50 years of progress in the use of telemedicine to improve patient outcomes in rural communities, systemic implementation barriers continue to isolate the communities at greatest need from taking full advantage of its benefits.^23^ Increasingly, these barriers are not exclusively technological, but rather are sociotechnical. That is, they result from the challenges associated with aligning technology investments effectively with clinical goals, user needs, administrative exigencies, and enabling policies to make the necessary change in rural America.^24^ In 2017, NCI, FCC, the UK MCC, UCSD, and Amgen formed a partnership dedicated to the goal of removing those barriers in rural Appalachia. A lesson learned from the collaboration is that if change is to be successful, localized, and swift it must originate in parallel from the communities themselves—not centrally with a top-down approach. Out of this understanding, project L.A.U.N.C.H. was born. The L.A.U.N.C.H. Collaborative welcomes the *JAH* and its readership to “barn-raise” with us in a goal to improve patients’ lives through improved broadband infrastructure (including access and adoption) and digital communications in rural America.

## Footnotes

a The Connect2Health^FCC^ Task Force, led and chaired by Deputy General Counsel Michele Ellison, is a senior-level, multi-disciplinary Task Force established by the FCC to move the needle on broadband and advanced healthcare technologies. The internal Task Force team, which includes attorneys, clinicians, data scientists and health IT experts, is charged with exploring the intersection of broadband, advanced technology, and health; and provides expertise and policy recommendations to the agency. The Task Force works to ensure that the FCC stays ahead of the health technology curve while also helping to bridge the digital divide in health and other sectors.

b The connectivity aspect of the project was borne by the recognition that health care is no longer just provided within the “four walls” of a hospital, clinic, or physician’s office; instead, mobile, wearable, ingestible and remote monitoring devices and systems—all enabled by broadband—allow health care to happen anytime or anywhere a patient needs and wants it. Cancer is a valuable use case to improve broadband access and adoption in rural Appalachia generally, thereby better ensuring that promising connected care solutions are available and accessible in this region, as well as other underserved areas of the country.

c It should be noted that the L.A.U.N.C.H. Collaborative, built on recommendations from the President’s Cancer Panel Report, pre-dated the formal granting programs authorized under the Beau Biden Cancer Moonshot segment of the 2016 21st Century Cures Act. Its funding was provided out of the partners’ operational resources to explore the value of a multi-agency, public-private collaboration.
